# Carpal tunnel syndrome and its associated factors among computer user bankers in South Gondar Zone, Northwest Ethiopia, 2021: a cross sectional study

**DOI:** 10.1186/s12891-023-06918-5

**Published:** 2023-10-19

**Authors:** Biruk Demissie, Chalachew Yenew, Alelign Alemu, Berihun Bantie, Bickes Wube Sume, Yikeber Argachew Deml, Eniyew Tegegne

**Affiliations:** 1 Department of Environmental Health, College of Health Sciences, Debre Tabor University, Debre Tabor, Ethiopia; 2 Department of Environmental Health and Hygiene, Bonn University Hospital, Bonn, Germany; 3 Department of Comprehensive Nursing, College of Health Sciences, Debre Tabor University, Debre Tabor, Ethiopia; 4 Department of Biomedical Sciences, School of Medicine, Debre Markos University, Debre Markos, Ethiopia; 5 Department of Environmental Health, College of Health Sciences, Debre Markos University, Debre Markos, Ethiopia

**Keywords:** Carpal tunnel syndrome, Banker, Factor, Computer user, Ethiopia

## Abstract

**Introduction:**

The use of information devices like computers is skyrocketed in recent years, leading injuries. Carpal Tunnel Syndrome is a leading cause of upper extremity MSDs specially to banking workers. Hence, this paper was intended to highlight its magnitude associated factors in the study area.

**Methods and materials:**

Institutional based cross-sectional study was conducted from September 13, 2021 to October 09, 2021. A total of 422 private and government owned computer user bankers were participated. Simple random sampling technique was used to select the study participants. Data were collected using Durkan’s compression test, flexion and compression test, Phalen’s test, and Tinel’s test. Multivariable logistic regression model was used to investigate the relationship between predictors and Carpal Tunnel Syndrome. P-value less than 0.05 was considered to declare as a significant and Adjusted Odds Ration for strength association between risk factors and Carpal Tunnel Syndrome.

**Result:**

Among 422 participants, the annual prevalence of CTS was 11.7%. Being smoker [AOR: 4.2; 95% CI: 1.76–10.26], having > 5-year work experience [AOR: 7.98; 95% CI: 3.7-17.33], movement repetition [AOR: 3.9; 95% CI: 1.66–9.4] and lack of ergonomics training [AOR: 5.2; 95% CI: 2.8–9.5] were independently associated risk factors to Carpal Tunnel Syndrome.

**Conclusion:**

Carpal Tunnel Syndrome was high (11.7%) among bankers in this study area. Carpal Tunnel Syndrome was predicted by smoking, length of employment, movement repetition, and not received ergonomics training. Therefore, fore the banking industry, it would be better to maintain strict follow-up and provision of ergonomics training.

## Introduction

The use of information and communication devices like desktops, laptops, and palmtops has risen rapidly in recent years, resulting in an increase in upper extremity injuries or musculoskeletal disorders (MSDs) [1]. When the workload exceeds the load-bearing capacity of the musculoskeletal systems, the problem arises [2]. Some studies discovered that there is a link between computer use and musculoskeletal symptoms [3–9]. The prevalence of work-related MSDs is rapidly increasing as technology advances [10]. There is a significant association between hours of computer use and hand/wrist disorders. Newspaper employees working at least 8 h per day were 3.3 times higher risk for developing MSDs than those using a computer for less than 8 h [4].

Among MSDs, Carpal Tunnel Syndrome (CTS) is commonly reported in computer users Office workers, particularly typists and data entry clerks, have been found to experience a high incidence of CTS. It is the leading cause of disability and a significant financial burden on both employees and employers. One third of the population misses more than 3 months per year due to CTS [11]. There is no consistent result regarding on the association between computer work and CTS. Some studies reviled that there is a link between computer work and CTS [12], whereas many other studies reported no association b/n computer work and CTS [13, 14]. On the other hand CTS has been found to become more common among office workers, particularly workers working with computer [10, 15]. A survey of workers who were classified as frequent computer users revealed that 10.5% of them fit the clinical criteria for CTS, while 29.6% of them had hand paresthesia [3, 14, 16].

Nerve injuries in the upper limbs, particularly CTS, have resulted from the frequent use of computer devices(16) by compression of the median nerve as it passes through the carpal tunnel as a result of repetitive movement and dynamic muscle shrinkage in the upper limb. It’s most commonly linked to work-related activities. Increased pressure in the carpal tunnel causes CTS [14, 17]. Workers who have jobs that require a lot of physical exertion, such as jobs that require a lot of forceful and repetitive hand movements, as well as awkward posture, are more likely to develop CTS [14].

Respondents with longer workdays have higher rates of CTS than those with shorter workdays. Workers who have years of service > 8 have 8.9 times higher risk of experiencing CTS than workers who have < 8 years of service. Mechanical workload, exposure duration, repetition frequency, working posture, obesity, overweight, female gender, advancing age, and cigarette smoking are all factors linked to MSDs [2, 14, 18–20]. However, in most previously published studies, the association between the dependent and independent variables was not consistent [14, 21]. The magnitude of CTS in Ethiopia is poorly understood, and it has given little attention. As a result, data were not available on it. Therefore, this study was aimed to determine the magnitude and its associated factors of CTS among computer user bankers in South Gondar zone, North West Ethiopia.

## Materials and methods

### Study area, period, and design

An institution-based cross-sectional study was conducted among computer user bankers working in South Gondar zone banks from September 13, 2021 to October 09, 2021. There were 21 governmental and 14 private owned banks and a total of 674 computer user bankers. Among these, 500 were workers of government owned and 174 were working in private owned banks.

### Source and study population

All computer user bankers working in South Gondar zone banks were considered as the source population, while all computer user bankers working in the randomly selected bank branches were taken as the study population.

### Eligibility criteria

All computer user bankers having one or more years of work experience were included in the study; whereas those with a history of MSDs, pregnancy, and critically ill bankers were excluded.

### Sample size determination and sampling technique

Single population proportion formula was used to calculate sample size by using 5% margin of error, 95% confidence intervals, P = 50% expected prevalence of WMSD in South Gondar Zone, and the final sample size (n) was 422 after adding a 10% non-response rate. A simple random sampling technique was used to select three private and one government owned banks, and 422 participants were proportionally allocated to private and government-owned banks. As a result, a total of 313 (from 500) bankers from government bank and 109 (from 174) bankers were proportionally allocated to government and private worker. Three hundred three participants were selected by lottery method from the total of government owned bank workers and likewise one hundred nine were selected randomly to include in to the study from the total of private owned bank workers.

### Data collection tools and procedures

Symptoms on the Katz hand diagram and a physical examination were used to diagnose CTS. All participants underwent durkan’s compression test, flexion and compression test, Phalen’s test, and Tinel’s test [22–26]. A pre-tested structured standardized questioner was used [3, 18, 27, 28]. The questionnaire was divided into three sections as sociodemographic, occupational, and personal factors. Data collection was carried out by two bachelors of science in physiotherapy, and three environmental and occupational health professionals. The process was supervised by two master degree holder environmental health professionals.

### Operational definitions

#### Computer user bankers

any bank workers who performs their activity using computer.

#### Cigarette smoking

an employee who smokes cigarette daily (at least one cigarette per day) for at least one year was considered as a smoker.

#### Body Mass Index (BMI)

< 18.5 is considered as underweight, 18.5– 24.9 is considered as normal, 25.0–29.9 considered as overweight, and ≥ 30.0 considered as obese [29].

#### Physical Exercise

Exercising or participating in any sport activity for at least 150 min of moderate-intensity aerobic physical activity per week, or 75 min of vigorous-intensity aerobic physical activity per week [30].

#### Alcohol consumption

an employee who drinks at least five drinks per week for men and two drinks per week for women for at least one year [31].

#### Repetitive work or Movement

A high repetitive job is defined as one which involves the repetitive use of awkward wrist movements lasting less than 30s or when more than 50% of work time is spent performing tasks that involve repetitive awkward wrist movements [32].

#### Carpal tunnel syndrome

Is pain or paraesthesia numbness or tingling on the anterior surface of the index, middle or radial half of the ring finger [33].

#### Tinel’s sign

In this test, the examiner taps lightly over the site of the median nerve at the distal wrist crease. Development of tingling or discomfort in the fingers supplied by the median nerve constitutes a positive sign [34].

#### Phalen’s test

It is performed the test by having the patient hold the forearm vertically with the elbows resting on the table and then allowing both hands to drop with complete wrist flexion for approximately one minute. The test is considered positive when paresthesia develops in less than one minute [35, 36].

### Data management and statistical analysis

The data were checked for consistency and completeness before being entered into Epi- info version 7 and exported to SPSS version 23. The data were presented in the form of frequency, percentage, mean, and standard deviation. Bivariate analysis was used, and variables with a p value of less than 0.25 were transferred and analysed using multivariate analysis irrespective of Crude Odds Ratio (COR) between one predictor with CTS. Multi-collinearity was checked using variance inflation factor. To control confounding variables; multivariate analysis was used to see the relationship and strength between predictors and outcome variables. P value less than 0.05 was used as the cut of point to decide statistically significance variables and the respective Adjusted Odds Ratio(AOR) was generated to measure the strength of association between risk factors and CTS.

### Data quality assurance

Standardized tools were adapted from various sources and written in English, then translated into the local language and back to English to ensure consistency. Data collectors and supervisors were received two days of training on data collection techniques, confidentiality, and the study’s objectives prior to data collection. Pretest was done in North Gondar banks using 5% of the study sample, and back then tool was modified accordingly.

### Result

#### Sociodemographic characteristics of the respondents

Data were collected from 422 computer user bankers and their age ranged from 21 to 51 years. Majority of the participants 65.2% (275) were under 30 ages. The mean age of the participants was 29.2, with a standard deviation (SD) of 9.1. 65.8 (278) participants were Male and 55% (232) were married. Majority of respondents 75.8% (320) had a bachelor’s degree and 80.8% (341) participants had a body mass index (BMI) of 18.5– 24.9(Table 1).

**Table 1:** Sociodemographic characteristics of computer user bankers in South Gondar Zone, North West Ethiopia, 2021 (n = 422)

Variables	Frequency (n)	Percent (%)
**Gender**		
Male Female	278 144	65.8 34.2
**Age**		
< 30 30–45 > 45	275 113 34	65.2 26.2 8.1
**Educational status**		
Diploma Degree MSc and above	59 320 43	14 75.8 10.2
**Marital status**		
Single Married	190 232	45 55
**BMI in Kg/m** ^**2**^		
Under weight (< 18.5) Normal (18.5-24.99) Over weight (25-29.99)	67 341 14	15.9 80.8 3.3
**Left-handed or right-handed**		
Right-handed Left-handed	404 18	95.7 4.3
**Designation**		
Clerical Assistant manager Manager Others	285 73 38 26	67.5 17.3 9 6.2

### Behavioural and work-related characteristics of the respondents

Among 422 participants 92.2% were not doing regular physical exercise, and majority of the participants were working in an awkward posture (positions of the body that deviate significantly from the neutral position while performing work activities which includes twisting, bending, reaching, pulling or lifting) (96.7%). Alcohol drinking and cigarette smoking was found in 27.7% and 11.1% of the participants, respectively. Furthermore, 39.6% of the participants had not undergone ergonomics training, and three-quarters (73%) of the participants had ≤ 5 years of work experience (Table 2).

**Table 2:** Behavioural and work-related characteristics computer user bankers in South Gondar Zone, North West Ethiopia, 2021 (n = 422)

Variables	Frequency	Percent
Cigarette smoking		
Yes No	47 375	11.1 88.9
Alcohol drinking		
Yes No	117 305	27.7 72.3
Physical exercise		
Yes No	33 389	7.8 92.2
Ergonomics training		
Yes No	167 255	39.6 60.4
Work experience in year		
1–5 > 5 Mean work experience in years	308 114 4.3 (SD 2.5)	73 27
Movement repetition		
Yes No	366 56	86.7 13.3
Working posture		
Good/Normal Poor/Awkward	14 408	3.3 96.7
Physical exercise		
Yes No	33 389	7.8 92.2
Working duration per day (in hours)		
≤ 8 > 8 Mean working duration per day in hours (SD)	374 48 8 (SD 2.2)	88.6 11.4
Duration of computer use per day (in hours)		
≤ 8 > 8 Mean computer use per day in hour (SD)	245 177 7.9(3.2)	58.1 41.9
Break		
Yes No	177 245	41.9 58.1
Type of bank		
Government Private	313 109	74.2 25.8

### The magnitude and associated factors of CTS

CTS was reported by 11.7% of the participants, with 13.7% of females and 7.6% of males. Additionally, 83.7% had a master’s degree or higher education and 14.6% were married. 37% of participants had five or more years of job experience, and 18.8% and 36.1% of participants were smokers and drinkers, respectively (Table 3).

**Table 3:** The magnitude of CTS among computer user bankers in South Gondar Zone, North West Ethiopia, 2021 (n = 422)

Variables	CTS		
	Yes	No	Total
**Sex**			
Male Female	38(13.6%) 11(7.6%)	240(86.3%) 133(92.4%)	278 144
**Age**			
< 30 30–45 ≥ 45	18 (6.5%) 16 (14.1%) 15 (44.1%)	257 (93.5%) 96 (84.9%) 19 (55.9%)	275 113 34
**BMI**			
Underweight Normal weight Overweight	11(16.4%) 37 (10.9%) 1 (7.1%)	56 (83.5%) 304 (89.1%) 13 (92.9%)	67 341 14
**Educational status**			
Diploma Degree MSc and above	7(11.9%) 37(11.6%) 5(11.6%)	52 (88.1%) 283 (88.4%) 38 (88.4%)	59 320 43
**Marital status**			
Married Single	34(14.7%) 15(7.9%)	198(85.3%) 175(92.1)	232 190
**Right or left-handed**			
Right Left	49 (12.1%) 0(0.0%)	355 (87.9%) 18 (100%)	404 18
**Alcohols drinking**			
Yes No	22(18.8%) 27(8.9%)	95 (81.2%) 278 (91.1%)	117 305
**Cigarette smoking**			
Yes No	16 (34%) 33 (8.8%)	31(66%) 342 (91.2%)	47 375
**Ergonomics training**			
Yes No	2(14.3%) 47(11.5%)	12(85.7%) 361(88.5%)	14 408
**Working posture**			
Good Poor	1 (7.1%) 48 (12.8%)	13 (92.9%) 360 (88.2%)	14 408
**Working experience**			
1–5 >5	14(4.5%) 35(30.7%)	294 (95.5%) 79 (69.3%)	308 114
**Movement repetition**			
Yes No	43(11.8%) 6(4.6%)	348 (88.2%) 125 (89.3%)	291 131
**Physical exercise**			
Yes No	1(3%) 48(12.3%)	32 (97%) 341 (87.7%)	33 389
**Working duration per day (in hours)**			
≤ 8 > 8	10 39	364 9	374 48
**Duration of computer use/day (in hours)**			
≤ 8 > 8	23 26	222 151	245 177
**Break**			
No Yes	15 34	230 143	245 177
**Type of bank**			
Governmental Private	20 29	293 80	313 109

### Factors associated with computer user bankers

In bivariate analysis female gender, older than 30 age, being single, alcohol drinking, cigarette smoking, having 5 or more years of work experience, movement repetition, not doing regular physical exercise, not received ergonomics training and using computer for more than eight hours per day were found to be associated with CTS, while in multivariate analysis, age greater than 30, having at least five years of work experience, using a computer for more than eight hours a day, repetitive movement, not having ergonomics training, and smoking were substantially associated with CTS.

Participants aged 30–35 years and older than 45 years of age were 6.5-fold [AOR: 6.5; 95% CI: 1.7–24.6] and 16.9 times [AOR: 7.9; 95% CI: 7.9–35.9] higher risk for CTS respectively, than those who aged less than 30 years. cigarette smokers were 4 times [AOR: 4.25; 95% CI: 1.76–10.26] more likely to develop CTS than non-smokers, and those who did not obtain ergonomics training were 5.2 times [AOR: 5.2; 95% CI: 2.8–9.5] more likely to have CTS than those who had. Participants who worked for more than 8 h on a computer were 5.2-fold [AOR: 5.2; 95% CI: 2.8–7.8] more likely to develop CTS than participants who worked for less than 8 h on a computer, and participants with 5 or more years of work experience were 7.98 times more likely [AOR: 7.98; 95% CI: 3.70-17.33] to develop CTS than participants with less than 5 years of work experience. Participants who repeated their wrist movements more frequently had a 5.4-times [AOR: 5.4; 95% CI: 1.29–8.96] higher risk of CTS than those who repeated them less frequently (Table 4).

**Table 4:** Associated factors of Carpal Tunnel Syndrome among computer user bankers (n = 422)

Variable	With CTS	Without CTS	COR (95%CI)	AOR (95%CI)	P - value
**Sex**					
Male	38 (13.6%)	240 (86.3%)	1	1	
Female	11 (7.6%0	133 (92.4%)	2.85 (1.85–4.05)	2.2(1.3–3.6)	0.31
**Age**					
< 30	18(6.5%)	257 (93.5%)	1	1	
30–45	16(14.1%)	96 (84.9%)	2.4 (1.8–3.89)	6.5(1.7–24.6)	0.006
≥ 45	15(44.1%)	19 (55.9%)	3.86 (1.34–5.219)	16.9(7.9–35.9)	0.000
**Educational status**					
Diploma	7(11.9%)	52(88.1%)	1		
Degree	37(11.6%)	283(88.4%)	0.6 (0.04–0.89)		
MSc and above	5(11.6%)	38(88.4%)	0.68 (0.23–098.2)		
**Marital status**					
Married	34(14.7%)	198(85.3%)	1		
Single	15(7.9%)	175(92.1)	1.99 (0.971–4.079)	1.94 (0.8–4.5)	0.11
**Alcohols drinking**					
Yes	22(18.8%)	95 (81.2%)	2.256 (1.14–4.48)	0.89 (0.52–1.5)	0.2
No	27(8.9%)	278 (91.1%)	1		
**Cigarette smoking**					
Yes	16(34%)	31(66%)	5.625 (2.56–12.34)	4.25 (1.76–10.26)	0.001^*^
No	33(8.8%)	342 (91.2%)	1	1	
**Ergonomics training**					
Yes	2(14.3%)	12 (85.7%)	1	1	
No	47(11.5%)	361(88.2%)	3.2 (2.1–4.9)	5.2 (2.8–9.5)	0.000^*^
**Working experience in year**					
< 5	14(4.5%)	294 (95.5%)	1	1	
≥ 5	35(30.7%)	79 (69.3%)	9.5 (4.49–20.19)	7.98 (3.7-17.33)	0.001^*^
**Movement repetition**					
Yes	43(11.8%)	348 (88.2%)	3.4 (1.29–8.96)	5.4(1.29–8.96)	0.000^*^
No	6(4.6%)	125 (89.3%)	1	1	
**Physical exercise**					
Yes	1(3%)	32 (97%)	1	1	
No	48(12.3%)	341(87.7%)	2.5 (1.6–3.9)	1.9 (0.9–2.4)	0.2
**Working duration per day (in hours)**					
**≤ 8**	10 (2.4%)	364 (86.3%)	1		
**> 8**	39 (9.2%)	9 (2.1%)	3.06 (1.4–6.5)		
**Duration of computer use/day (in hours)**					
**≤ 8**	23 (5.4%)	222 (52.6%)	1	1	
**> 8**	26 (6.2%)	151 (35.8%)	4.072(2.6–6.3)	5.2 (2.8–7.86)	0.027^*^
**Break**					
**No**	15 (3.6%)	230 (54.5%)	0.95(0.6–1.4)		
**Yes**	34 (8%)	143 (33.9%)	1		
**Type of bank**					
**Governmental**	20 (4.7%)	293 (69.4%)	1		
**Private**	29 (6.9%)	80 (19%)	0.55(0.35–0.8)		

*p ≤ 0.05

## Discussion

Long-term computer use has been shown to increase the risk of CTS, and there is a strong link between computer use and CTS [37]. In this study, the annual prevalence of CTS among computer user bankers was 11.7% (49/422). This showed that one in every nine computer user bankers experienced CTS each year. This finding was consistent with the result found in Kuwait [18], Malaysia [19], China [38], and India [3]. They found that 13.1%, 16.5% and 18.7% of CTS among computer user bankers respectively. However, the highest results were reported from USA (29.6%) [3], among touchscreen users of Majmaah University (34.2%) [39], Saudi Arabia (50%) [10], Abha City (51.1%) [40] and Pakistan (61.5%) [37], and lowest result was reported in Denmark (5.5%) [13]. This discrepancy may result due to different in study period, working conditions and workloads between nations. Their risk of CTS may increase as a result of longer working hours or years.

The current study revealed that, the prevalence of CTS was higher among participants aged 30–45 and > 45 years had 6.5- and 16.9-fold higher risk of CTS than under 30 years. This finding was consistent with findings from Kuwait [18], India [3], Pakistan [37] and Iraq [41]. Increased the severity of CTS with increasing age [42, 43]. Several studies have found that CTS in elderly people is associated with more severe motor and sensory axon loss, exclusively diurnal paresthesias, and thenar muscle atrophy [43]. This could occur as a result of aging, which has a significant effect on a number of peripheral nerve features, including decreased production of the main myelin proteins, demyelination, age-related degenerative changes to muscles, tendons, ligaments, or joints [44], loss of myelinated and unmyelinated fibers, and delayed regeneration [45].

In this study, respondents who had worked for 5 years or longer were 7.9 times at higher risk of CTS than those who had worked less than 5 years. These findings were consistent with the study done in India, Indonesia, and Bhakti Kencana, which showed that bankers who used computers were 3.3, 8.2, and 2.1 times more likely to develop CTS respectively [3, 27, 46] [24] [19]. Another study conducted in Iraq found that computer users who had greater job experience were more likely to develop CTS than those who had less expertise [41]. This might be directly related with the age of the participants because people tend to work more as they become older. Shift work or work rotation will be better if implemented to reduce the burden of CTS and related MSDs.

In the present study, participants who used computers for more than 8 h per day had a 5.2-fold higher risk of getting CTS than those who used computers for less than 8 h per day. This is in line with the study done in India and Indonesia, they found 4.9 [3] and 6.14 [46] times higher risk for CTS respectively. The risk of the problem probably increased by repeatedly performing the same tasks in the same working posture for an extended period of time.

The risk of CTS among participants who smoked cigarettes was 4.2 times higher than that of non-smokers. This is consistent with an Indian study that discovered smokers had a 1.6 times greater risk of CTS than non-smokers. A Danish study also confirmed that being smoker was significantly associated with CTS [3, 13, 47]. Cigarette smoking is associated to decreased blood flow, oxidative stress, and systemic inflammation, which may deteriorate peripheral nerves and make them more susceptible to compression neuropathies and it may also increase the risk of median nerve damage through toxic effects [48]. The bank industry need to strive to aware risks of smoking and work to rehabilitate workers with the habit of smoking by filling their underlying reasons smoking.

Participants who didn’t receive ergonomics training were 5.2 times more likely to develop CTS than those who did. According to earlier studies on the efficiency of ergonomic training reported there is a positive association between ergonomics training and MSDs symptom reduction. The studies confirmed that ergonomics training improves worker knowledge, work station habit, promotes safe working practices, and resulted in changes in the hand/wrist posture when using computers [49–53]. Regarding the position of the keyboard significant improvements were found among trained participants than untrained [51–53]. Ergonomics training may assist them to have important information about the safety measures to take while working, the severity of symptoms and functional status of carpal tunnel syndrome can be reduced [54].

Participants who performed more than 30 repetitive movements within one minute increased the risk of CTS by 5.4 times compared to those who performed less than 30 repetitions in one minute. This finding is supported by the result found in Bhakti Kencana, where it was discovered that participants who experienced > 30x in a minute had a 1.8 times higher risk of having CTS [27, 55]. Inside the wrist is a sort of archway - the carpal tunnel. This tunnel is where the tendon and nerve responsible for finger flexion pass. The tissues inside the carpal tunnel might get inflamed and swollen if the wrists repeatedly in the same manner. The nerve is then compressed in the carpal tunnel, which results in the numbness, tingling, and other Carpal Tunnel Syndrome symptoms [56].

## Conclusion

In this study area, the magnitude of CTS was high. Respondents with history of cigarette smoking, being older, long duration of employment and longer duration of working hours, and luck of ergonomics training were significantly increased the risk for CTS. The bank sector should have strict follow up and should provide ergonomics training for new bankers.

### Limitation of the study

The study is solely a cross sectional design which cannot ascertain temporal relationship of between predictor variables and CTS.

## Data Availability

All data gathered and analyzed during this study are available from the corresponding author at reasonable requisite.

## Abbreviations and Acronyms

CTS: Carpal Tunnel Syndrome
MSDs: Musculoskeletal Disorders
COR: Crude Odds Ratio
AOR: Adjusted Odds Ratio
